# COMPANION: development of a patient-centred complexity and casemix classification for adult palliative care patients based on needs and resource use – a protocol for a cross-sectional multi-centre study

**DOI:** 10.1186/s12904-021-00897-x

**Published:** 2022-02-04

**Authors:** Farina Hodiamont, Caroline Schatz, Daniela Gesell, Reiner Leidl, Anne-Laure Boulesteix, Friedemann Nauck, Julia Wikert, Maximiliane Jansky, Steven Kranz, Claudia Bausewein

**Affiliations:** 1 Department of Palliative Medicine, LMU University Hospital, Munich, Germany; 2 Helmholtz Zentrum München, Institute of Health Economics and Health Care Management, Munich, Germany; 3 Ludwig-Maximilians-Universität München, LMU Munich School of Management, Institute of Health Economics and Health Care Management, Munich, Germany; 4 Ludwig-Maximilians-Universität München, Institute for Medical Information Processing, Biometry and Epidemiology (IBE), Munich, Germany; 5 Clinic for Palliative Medicine, University Medical Center, Göttingen, Germany; 6 German Association for Palliative Medicine, Berlin, Germany

**Keywords:** Palliative care, End of life care, Casemix classification, Cost calculation, CART analysis

## Abstract

**Background:**

A casemix classification based on patients’ needs can serve to better describe the patient group in palliative care and thus help to develop adequate future care structures and enable national benchmarking and quality control. However, in Germany, there is no such an evidence-based system to differentiate the complexity of patients’ needs in palliative care. Therefore, the study aims to develop a patient-oriented, nationally applicable complexity and casemix classification for adult palliative care patients in Germany.

**Methods:**

COMPANION is a mixed-methods study with data derived from three subprojects. Subproject 1: Prospective, cross-sectional multi-centre study collecting data on patients’ needs which reflect the complexity of the respective patient situation, as well as data on resources that are required to meet these needs in specialist palliative care units, palliative care advisory teams, and specialist palliative home care. Subproject 2: Qualitative study including the development of a literature-based preliminary list of characteristics, expert interviews, and a focus group to develop a taxonomy for specialist palliative care models. Subproject 3: Multi-centre costing study based on resource data from subproject 1 and data of study centres. Data and results from the three subprojects will inform each other and form the basis for the development of the casemix classification. Ultimately, the casemix classification will be developed by applying Classification and Regression Tree (CART) analyses using patient and complexity data from subproject 1 and patient-related cost data from subproject 3.

**Discussion:**

This is the first multi-centre costing study that integrates the structure and process characteristics of different palliative care settings in Germany with individual patient care. The mixed methods design and variety of included data allow for the development of a casemix classification that reflect on the complexity of the research subject. The consecutive inclusion of all patients cared for in participating study centres within the time of data collection allows for a comprehensive description of palliative care patients and their needs. A limiting factor is that data will be collected at least partly during the COVID-19 pandemic and potential impact of the pandemic on health care and the research topic cannot be excluded.

**Trial registration:**

German Register for Clinical Studies trial registration number: DRKS00020517.

## Background

Palliative care has become an integral part of the German health care system and is experiencing an enormous increase in services, both in the community and inpatient setting. There are currently more than 350 palliative care units, 360 specialist palliative home care teams, and 70 palliative care advisory teams in Germany [1]. According to estimates, around 60,000 patients are treated in palliative care units and around 65,000 in the home care setting receive specialist palliative care annually, but hardly any valid data are available on the precise number of patients [2]. The need for palliative care (general and specialist) in Germany is estimated between 700,000 and 850,000 (about 1% of the total population) – with a rising trend [3]. Due to the demographic change and the increasing number of elderly, comorbid people, the German health care system will face an economic challenge that reinforces the necessity of a needs-oriented health care approach – especially for palliative care.

The intensity of individual symptoms or psychosocial, spiritual, or ethical problems, as well as their interaction and simultaneous occurrence, determine the complexity of the patient’s situation [4, 5]. The evidence and consensus based guideline, “Palliative medicine for patients with incurable cancer”, recommends determining the need for specialist palliative care in relation to the complexity of patients’ needs [6]. Complexity is determined by the number, type, and interaction of various physical, psychological, social, spiritual, and practical patient and family needs [6].

So far, there is no valid evidence-based system in Germany to determine the complexity of patients’ needs in palliative care and to screen patients according to their needs for an indication of specialist palliative care. The only system currently used in Germany to differentiate between patients is the Diagnosis Related Groups System (DRG system) which, being a commonly used cost reimbursement system in German hospitals, is mainly based on diagnoses and procedures. However, in palliative care, international data indicate that diagnoses are inappropriate indicators for the need of resources and the associated costs [3, 7]. First national data show that the same is valid for the German Health Care context. Studies demonstrate that diagnoses is not an indicator for resource use in Germany and that the German Diagnosis-Related Groups system is inadequate to describe resource needs and associated costs in palliative care [8, 9].

In Australia, a casemix classification, developed in the 1990s to assess the complexity of palliative care patients, was used to develop a financing system for palliative care - beyond the DRG system. Based on 5000 patient data records and associated costs, the Australian casemix classification identified palliative care phase, age, functional status, and severity of symptoms and other problems as important “cost drivers” and therefore defined them as casemix criteria [3, 10]. A similar classification is currently being developed and tested in England [11].

For Germany, a corresponding classification can serve to better describe the patient group in palliative care and thus help to develop adequate future care structures. A casemix classification used throughout Germany would also enable national benchmarking and quality control. Disparities in outcomes between different care institutions, caused by heterogeneous patient groups treated, can be eliminated by adapting the outcomes to the casemix criteria to trace back the remaining differences to differences in care models or care itself. Finally, such a casemix classification could form the basis for a new financing model for palliative care, which accounts for the differences in care costs and reflects the costs of the various palliative care institutions. It cannot be excluded that differences in the model of care, such as structure and process characteristics, have an impact on calculated costs on the institutional and/or patient level. A cost calculation should, therefore, account for potential differences.

Due to existing differences in the structure and financing of the national health care systems, the available and currently emerging systems from Australia and England can only be transferred to Germany to a limited extent.

Therefore, the study aims to develop a patient-oriented, nationally applicable complexity and casemix classification for adult palliative care patients in Germany. Data from three subprojects will inform the development of the casemix classification.

- Subproject 1 aims to describe patients cared for in specialist palliative care in Germany and patients’ needs that reflect the complexity of the respective patient situation, as well as the resources used to meet/address these needs.
- Subproject 2 aims to develop a taxonomy for specialist palliative care models in Germany.
- Subproject 3 aims to identify patient related cost drivers for palliative care and develop a cost calculation for specialist palliative care in Germany.

## Methods/design

To ensure a high scientific standard and transparency, the reporting of this study protocol and the study results of each subproject will follow the “Strengthening the Reporting of Observational Studies in Epidemiology “(STROBE) guideline, the “Transparent Reporting of a multivariable prediction model for Individual Prognosis or Diagnosis” (TRIPOD) statement and the “Consolidated criteria for reporting qualitative research “(COREQ) Checklist [12–14]. The study is registered at the German Register for Clinical Studies (DRKS trial registration number: DRKS00020517). Subproject 2 is a qualitative study among professionals and, therefore, not registered as a clinical study.

### Study design

**Fig. 1:**
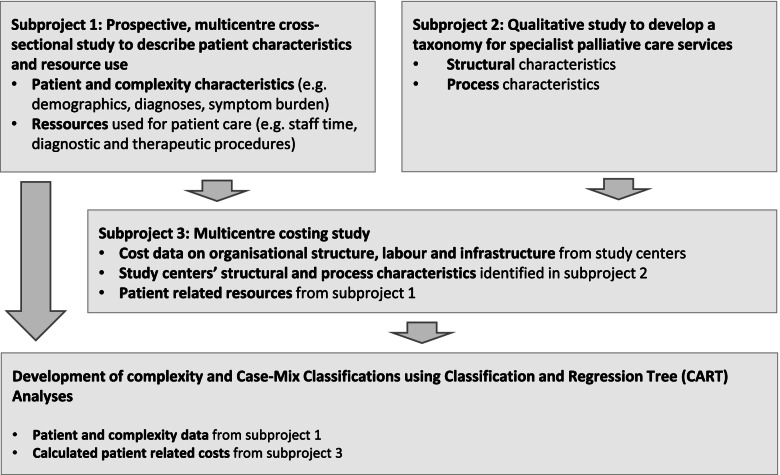
Proceeding subproject 1–3

COMPANION is a mixed methods study that aims to develop a casemix classification for adult palliative care patients in Germany. Data and results from subprojects 1–3 will inform each other and form the basis for the development of the casemix classification (see Fig. 1).

- **Subproject 1:** Prospective, cross-sectional multi-centre study collecting data on patients’ needs which reflect the complexity of the respective patient situation, as well as data on resources that are used to meet these needs.
- **Subproject 2:** Qualitative study including the development of a literature-based preliminary list of characteristics, identification of relevant structure and process characteristics by expert interviews, and a focus group to develop the taxonomy based on identified characteristics.
- **Subproject 3:** Multi-centre costing study based on data of resources from subproject 1 and retrospective data of study centres following predefined criteria. The methodology of collecting data on resource use and cost calculation on patient level were tested and further developed through a feasibility study conducted in the Department of Palliative Medicine at the Munich University Hospital in 2016 [9].

### Setting

#### Subproject 1 - cross-sectional multi-Centre study

Patient data is expected to be collected in 28 German specialist palliative care services. Services are located all over Germany and cover inpatient settings (palliative care units, palliative care advisory teams) as well as specialist palliative home care. Institutions will be selected according to predefined criteria (urban/rural spatial structure, university affiliation of the service, location of service regarding deprivation index of the region, and regional population share aged > 65 years).

#### Subproject 2: qualitative study

The expert interviews and the focus group will be conducted as end-to-end encrypted web conferences. Date, time, and communication medium are preferably determined by the interview partner.

#### Subproject 3: multi-centre costing study

Data will be collected for all study centres reporting cost and structural data as requested in the cross-sectional multi-centre study in subproject 1.

### Participants

#### Subproject 1 – cross-sectional study

Consecutive inclusion of all patients treated in the participating study centres (palliative care unit, palliative care advisory team in the hospital, specialist palliative home care) during the data collection period. Data will be collected for each patient over the course of an entire episode of care, which comprises the time between admission to a certain specialist palliative care setting and the termination of care in the respective setting, induced by either change of care setting or the patient’s death.

#### Subproject 2: qualitative study

Participants for both interviews and focus group are selected based on their in-depth insights into the organisation of palliative care structures in Germany. Experts are included from the above mentioned three specialist care settings.

#### Subproject 3: multi-centre costing study

Cost accounting data are collected for the entire year of 2019 to consider seasonal and cyclical fluctuations with all patients treated in 2019 in either of the palliative care settings.

### Data collection

#### Subproject 1: cross-sectional study

Data collection is scheduled from April 2021 to August 2022. At each study site, data will be collected over a period of 3 months. Continuous support and feedback provided by the research team will contribute to high data quality. Three types of data will be collected by clinical staff: (a) data on patients’ needs reflecting complexity, (b) patient demographics and diagnoses, and (c) data on resource use on patient level. Collected data will be processed exclusively in anonymised form. Since data can accordingly not be assigned to a natural person, the patients’ consent will not be required.

- (a) Data on patients’ needs: Measurement instruments

Based on the results of a qualitative pre-study to identify factors describing complexity of palliative care situations [5], and the Australian and the English casemix studies [10, 11], the following measurement instruments are used for the assessments of patient needs:

- *Integrated Palliative care Outcome Scale (IPOS)* [15]: The IPOS reflects the patient’s symptom burden and palliative care concerns using a 5-point Likert scale. The measure covers physical symptoms, psycho-social burden, family needs, and practical problems.
- *Palliative Care Problem Severity Score (PCPSS)* [16]: The PCPSS is a short assessment tool used to map the severity of symptoms and problems of patients and their relatives using a 4-point Likert scale. The assessment covers four domains: pain, other symptoms, psychological/spiritual, and family/carer problems. For the use in this study, the domains agitation and disorientation will be added.
- *Australian Karnofsky Performance Score (AKPS)* [17]: The AKPS uses 11 levels to map the functional status or a patient’s ability to perform daily activities.
- *20-Point modified Barthel Index* [18]: The Barthel Index records the functional status of a patient. It is used to systematically assess the ability of independent living or the need for care.
- *Palliative care phase* [19]: The palliative care phase is a holistic clinical assessment reflecting the needs of patients and their family members and caregivers. Five clinically significant phases can be differentiated (stable, unstable, deteriorating, terminal, and bereavement).

**Fig. 2:**
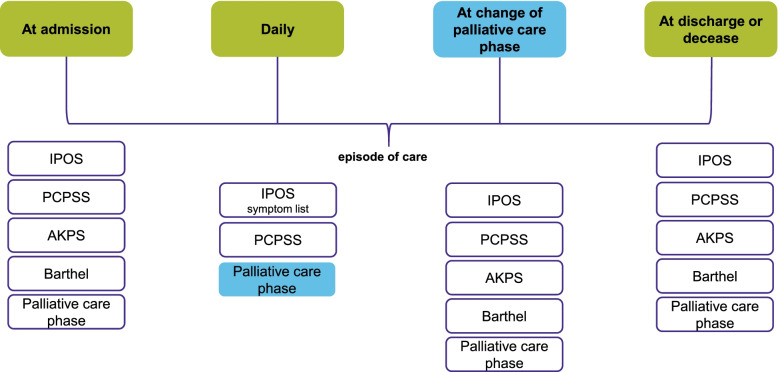
Time points and assessments

*Frequency of assessments*: A short version of the IPOS (covering physical symptoms), the PCPSS, and palliative care phase are assessed daily in the palliative care units and at every patient contact (face-to-face or by phone), maximum once a day, in specialist palliative home care as well as palliative care advisory teams (see Fig. 2). A change in the palliative care phase will trigger the assessment of the whole IPOS, PCPSS, AKPS, and Barthel Index.

- (b) Patient demographics, diagnoses, and other patient characteristics

In addition, routine and sociodemographic data on the included patients are collected, such as main diagnoses, time since first diagnosis, type of discharge, age, gender, living situation (in the home care setting), date of admission and discharge, and whether the person is a native German speaker.

- (c) Resources used on patient level

Staff resources: Staff time used to care for a patient and their relatives will be recorded in minutes (by five-minute intervals), subdivided by professional group.

Data is collected electronically and, where possible, will be integrated into the existing electronic patient records used for clinical documentation in the study centres. Staff time already routinely documented by professional groups involved in patient care will be completed by time data not routinely documented. The latter mainly applies to nursing hours. Which form of complementary time documentation is chosen depends on the internal documentation, IT conditions, and routines of the participating teams.

The unit for data collection is “patient contact” representing the smallest collectable data level. “Patient contact” is to be understood as every single contact between either clinical staff and patient or relatives, or patient-related discussion in team meetings and contacts to other professionals. Data on patient contacts will later be aggregated into “episodes of care”.

#### Subproject 2: qualitative study

Semi-structured expert interviews: The interview guide is developed by the project team following the four-step process suggested by Kalio et al. [20]. Over the course of the interviews, experts are invited to discuss suitable characteristics for the differentiation of specialist palliative care models in Germany. Subsequently, the interviewees are presented a preliminarily developed list of characteristics for the above-mentioned purpose. This list is based on an elaboration of the characteristics in the German “Guide for Hospice and Palliative Care” (www.wegweiser-hospiz-palliativmedizin.de) which is a web-based platform set up and run by the German Association for Palliative Medicine. It offers a voluntary registration of palliative care services and has been complemented in accordance with international studies [21–23] as well as the study team’s earlier work [5]. The experts are asked to comment on the suitability of the listed criteria for differentiating care models and to supplement the list as needed.

Focus group: Prepared results from the expert interviews in the form of a preliminary taxonomy will represent the basis for discussion in the focus group. The focus group will follow a guiding question and semi-structured interview guide.

#### Subproject 3: multi-centre costing study

Cost data will be collected on institutional or department level. Based on relevant international studies [10, 24, 25] and the study group’s preparatory work [9], a template with required information for preparing the cost calculation is sent to participating study centres. Generally, aggregated values at institutional or departmental level are requested, in standardised format for the three care settings. All relevant cost categories from the cost data supplying centres, which are expected to be recorded in the information systems of the institutions, are included. Beyond financial data, structural information is requested, especially the number of employees, subdivided into occupational groups. Additionally, structural data, like the number of patient contacts and nursing days for each patient, as well as the treatment cases, are collected. The predefined template and guideline aim to form the basis for a comparable assessment of resource use across the participating institutions. Regarding the different sizes of included centres, adjustments are made based on the number of patients. Data transfers take place directly between the evaluating centre (Helmholtz Zentrum München) and the individual study centres.

### Sample size calculation

#### Subproject 1: cross sectional study

Based on the pilot study realised in the Department of Palliative Medicine at the Munich University Hospital [8] and unpublished data from England (Prof. Murtagh, personal communication), about 25 variables are expected for the classification. The aggregated unit for data collection is “episode of care”, defined as the time of contact between patient and provider in a setting (e.g., inpatient stay, time at home) [7, 9]. There are no established methods to calculate the required sample size for the intended analyses based on Classification and Regression Trees (CART). Thus, the sample size is chosen on the basis of the “10 observations per variable” rule of thumb (which has been controversially discussed in the context of regression analysis but can be used to determine a rough order of magnitude in the context of CART analysis), yielding a number of approximately 250 episodes needed for each setting.

To obtain reliable, undistorted estimates for the prediction accuracy of the derived casemix classification, an independent data set including 250 episodes from each setting is used for validation. Based on the data from the Australian study, 25% of incomplete data sets are expected [10]. Up to 10% of episodes may be cost outliers (episodes with unusually high or low costs) that should be removed from the development of the casemix classification [9] to optimise the classification’s predictive validity for the majority of patients.

The final sample for each setting is (250 + 250)/(1–0.25-0.1) ≈ 770 per setting, leading to a total of 3 × 770 = 2310 episodes. Due to the novelty of the study for the German context, an interim analysis with the first 100 episodes per setting will be conducted. This is to estimate the percentage of incompletely documented episodes and outliers. If necessary, the total sample will be recalculated.

#### Subproject 2: qualitative study

Interview study: A total of about 8–10 expert interviews are conducted. In about three interview rounds of 3–4 interviews, the list of characteristics is discussed and adjusted based on the results. The modified list of characteristics will then be subject for the subsequent interviews.

Focus group: Following the analysis of expert interviews results, a focus group with 8–10 experts is conducted.

#### Subproject 3: multi-Centre costing study

As national standards for cost accounting for specialist palliative care do not exist in Germany, a sample size calculation is not possible. Instead of sampling, the study will provide a first collection of cost and structural data for comparative cost calculation across organisations. These costs are applied for the sample size described in subproject 1.

### Data analysis

#### Subproject 1: cross sectional study

Descriptive statistical analyses are conducted to describe the study population and resource use, as well as distribution of possible casemix criteria for comparison with existing data on episodes, palliative care phase, and other criteria describing complexity from the English and Australian studies [10, 11].

#### Subproject 2: qualitative study

Interview transcripts and field notes from the expert interviews are analysed using content-analytical techniques, described by Mayring and Kuckartz [26–28]. The software MAXQDA is used to facilitate data management and coding [29].

Codes are generated through a discursive process of analysis, including inductive and deductive coding to create categories by grouping conceptually similar themes. Deductive codes will emerge from the list of characteristics and predefine the code frame, while inductive codes deriving from the material enhance the results of analysis.

#### Subproject 3: multi-centre costing study

Bottom-up cost accounting methods combined with top-down approaches will be used to calculate costs at patient-level. The applied calculation method is based on relevant work from Australia and England [30, 31]. Personnel cost rates are calculated with staff time collected in subproject 1. This partly reflects the idea of time-driven activity-based costing [32]. Non-personnel costs are allocated on average, based on the InEK costing scheme (Institute for the Hospital Remuneration System) or facility specific cost calculations [33]. Results of this cost calculation, such as patient-level costs per day, per episode, and per palliative care phase, are used in the subsequent CART analyses.

##### Development of the casemix classification

To derive an easy-to-use and intuitive patient-oriented national complexity and casemix classification for adult palliative care patients in Germany, “Classification and Regression Tree” (CART) analyses will be conducted. CART analyses will use data on patient complexity from subproject 1 as predictor variables and cost data on the patient level calculated in subproject 3 as the dependent variable that must be modelled. Such trees aim to provide accurate prediction models for the cost while remaining intuitive and inspired by medical decisions, which often result from the answers to a sequence of binary questions; see Strobl et al. for a gentle introduction to decision tree methodology [34].

Individual CART analyses will be realized for each setting. Several variants of trees will be considered, including the unbiased so-called “conditional inference” decision trees (40). The prediction accuracy of these models will be estimated and compared within training data sets using an internal cross-validation procedure (while the validation data sets remain untouched). Multivariable regression models will also be fitted as an alternative to trees.

The selection of the final models will be performed using the training data sets only and will account for the accuracy, stability, and plausibility of the involved predictor variables. With the aim of obtaining a reliable estimate of the prediction accuracy of the derived models, an independent validation of these final models will be carried out using the validation data sets following up-to-date methodological recommendations [13].

### Methods for reducing Bias

#### Subproject 1: cross sectional study

A first step in avoiding bias is to select the participating study centres based on predefined criteria. Since all data is collected by employees in the participating centres, all patients can be included regardless of whether they are able to participate in data collection. If this were not the case, the collected data would be subject to a fundamental systematic error. Consequently, this would limit conclusions about the complexity of palliative care against the background of data basis since specific patient groups, probably the most vulnerable patients (unconscious, not eligible to consent), would be excluded from the analysis. An intensive training of the professionals in the study centres, regular feedback, and support from the responsible project team aims to maximise data quality. A manual and staff trainings are supposed to optimise the data quality and reduce missing data [35]. Estimated non-responses and missing data were taken into account in the sample size calculation (based on the Australian study [10]). If data is missing or incoherent, causes are explored, and appropriate imputation methods are used.

#### Subproject 2: qualitative study

Participants will be recruited based on a predefined sampling frame aiming for the inclusion of all relevant professional groups. A literature-based list of characteristics serves as the basis for the expert interviews, while a preliminary taxonomy subsequently forms the basis for the focus group. Underpinning the list of characteristics and German experts’ perceptions with international literature will prevent a one-sided view on relevant characteristics. Furthermore, this approach will reduce the risk of neglecting any essential aspects while less relevant (international) characteristics will be withdrawn based on the experts’ experience.

#### Subproject 3: multi-centre costing study

Due to the heterogeneity of structural data and financial information across study centres, some bias cannot be excluded. To minimise this, the calculation method will combine bottom-up with top-down approaches in predefined criteria and will thus bridge the gap between study centres.

## Discussion

The presented study aims to develop a casemix classification for adult patients in Germany. This work is closely linked to related studies in Australia and the UK. Ideally, the developed casemix classifications result in similar criteria to describe the casemix of patients in specialist palliative care across countries. However, as health care systems differ widely, independent research must be conducted in the respective countries.

The realisation of the study faces several challenges. The outbreak of the COVID-19 pandemic occurred during the recruitment period of study centres and data collection for subprojects 1 and 3. Public life in Germany was drastically reduced whilst the health care system reached its limits. Palliative care services are severely affected, facing restrictions in seeing patients at home or in the hospital due to visiting restrictions. Relatives are not allowed to visit their loved ones in palliative care units as flexibly as usual. This may impact on the complexity of patient situations as staff times and resources must be reallocated. Even though research currently investigates care providers’ and patients’ experiences during the pandemic, it remains unclear how care provision and related factors (e.g., patient characteristics) changed over the course of the pandemic, and possible long-term effects on palliative care provision in Germany cannot be estimated with certainty. To improve the understanding of the results of the COMPANION study, we will take into account findings from other research conducted from some of the project partners, notwithstanding from the COMPANION project, such as analyses of the impact of the pandemic on the use of resources and cost data, as well as on the complexity of patient situations.

Also, recruitment of study centres and the preparation of data collection is largely affected by the pandemic situation. The focus of palliative care services currently lies on managing the challenges of the pandemic and therefore, the willingness to participate in such a research project is rather limited.

The yet unknown influence of the pandemic on processes, structures, costs, and patient characteristics in palliative care is not only a challenge but may also limit this study’s validity. We are aware of this potential limitation and will address it by factoring in results from current research into the realisation of the study as well as in the interpretation of findings.

## Data Availability

Data sharing is not applicable to this article as no datasets were generated or analysed yet.

## Abbreviations

CART: Classification and Regression Tree
DRG: Diagnosis Related Groups
IPOS: Integrated Palliative care Outcome Scale
PCPSS: Palliative Care Problem Severity Score
AKPS: Australian Karnofsky Performance Score
